# Burden of malaria is higher among children in an internal displacement camp compared to a neighbouring village in the Democratic Republic of the Congo

**DOI:** 10.1186/s12936-016-1479-z

**Published:** 2016-08-25

**Authors:** Rhianna Charchuk, Makelele Katsuva Jean Paul, Kasereka Masumbuko Claude, Stan Houston, Michael T. Hawkes

**Affiliations:** 1 School of Public Health, University of Alberta, Edmonton, Canada; 2 École de Santé Publique, Université de Lubumbashi, Lubumbashi, Democratic Republic of the Congo; 3 Université Catholique du Graben, Butembo, Democratic Republic of the Congo; 4 Department of Medicine, University of Alberta, Edmonton, Canada; 5 Department of Pediatrics, University of Alberta, Edmonton, Canada

**Keywords:** Democratic Republic of the Congo, Internally displaced persons, Children, Cross sectional study

## Abstract

**Background:**

In the Democratic Republic of the Congo (DRC), violent conflict has caused the displacement of millions of people into camps where they are exposed to poor living conditions and high rates of infectious diseases. Malaria, in particular, is a major cause of mortality in children under five; however, the burden of disease in displacement camps has not previously been described.

**Methods:**

Two cross-sectional surveys were performed. First, prevalence of *Plasmodium falciparum* antigenemia was measured in a random sample of 200 children living in a displacement camp and 200 children from a nearby village (control group). Second, the proportion of febrile illness attributable to malaria was measured in a study of 100 children from the displacement camp and 100 children from the control village presenting to the same health clinic with fever. All participants were tested for *P. falciparum* with a rapid diagnostic test and additional demographic data, clinical characteristics, and malaria risk factors were determined using a parental questionnaire.

**Results:**

In the community survey, children living in the displacement camp had a higher prevalence of *P. falciparum* infection (17 %) than controls (7.5 %) (OR 2.6; 95 % CI 1.3–4.1; P = 0.0095). In the clinic-based survey, the proportion of febrile illness attributable to malaria was higher among children from the displacement camp (78 %) than controls (39 %) (OR 5.5; 95 % CI 3.0–10.3; P < 0.001). Household bed net ownership and use was significantly lower in the displacement camp than control village in both surveys. Statistically significant differences in household wealth, maternal education, and exposure to community violence were also found.

**Conclusions:**

Population displacement due to violent conflict appears to be a risk factor for malaria, a major cause of child mortality. Children living in displacement camps are a relatively understudied population, but have a high burden of malaria, despite control programmes focused on bed net distribution.

**Electronic supplementary material:**

The online version of this article (doi:10.1186/s12936-016-1479-z) contains supplementary material, which is available to authorized users.

## Background

Following decades of violent conflict, the Democratic Republic of the Congo (DRC) faces social disruption and decimated health care infrastructure [1]. The DRC ranks 176 out of 188 countries in terms of human development index [2] and 88 % of the population lives on less than US$1.25 per day [3]. Extreme poverty, limited infrastructure, and violent conflict have led to high mortality rates, especially from infectious diseases [4]. Specifically, the DRC suffers from one of the highest incidences of malaria worldwide with 6 million cases annually and over forty thousand deaths in 2013 [5].

Population displacement due to violent conflict may be associated with an elevated risk of malaria. Internally displaced persons (IDPs) often live in large displacement camps where access to sanitation, safe water, food and healthcare may be limited [6], under which conditions malaria transmission may be elevated [7]. Relative to refugees, who cross international borders and may benefit from international attention and aid, IDPs may be more dependent on weak government services for basic necessities. In the DRC, chronic violent conflict between national military, numerous militias and rebel groups has forced large numbers of people to flee their homes, and forced them to leave behind property, possessions and income-generating opportunities [8]. Currently, the DRC has over 2.7 million IDPs [9].

Higher rates of infectious diseases have been reported among populations forcibly displaced by violent conflict [10, 11]. This increase may be caused by several factors such as poor living conditions in refugee and displacement camps, the movement of a non-immune population into an area with endemic disease and limited access to healthcare services. Elevated morbidity and mortality due to malaria has been documented among displaced populations in several settings, including Afghan refugees in the 1990s [10], a Kurdish refugee camp in Iraq [12], and an IDP camp in Uganda [13]. However, the burden of malaria among children in IDP camps in the DRC has not been previously reported, to our knowledge. Further studies are warranted to describe the burden of infectious diseases among IDPs, who represent a neglected group in global malaria control efforts [14, 15]. The malaria burden was evaluated in a Congolese population displaced by violent conflict, comparing children from an IDP camp to controls from a nearby village. We also explored putative determinants of malaria infection, such as bed net use, household wealth and mother’s education.

## Methods

### Study setting

This study was carried out in the IDP camp of Bilobilo, established near the village of Mubi, in Walikale district, North Kivu province, Eastern DRC. A map of the country and North Kivu province is shown in Additional file 1. This region of the country has faced years of violent conflict. Recently, in the spring of 2012, a group of armed Rwandan rebels caused the displacement of over 200,000 resident Congolese, leading to the establishment of the temporary IDP camp in this study [16]. A recent investigation of mortality in the Walikale region found a high crude mortality rate and that the main cause of death of children under five was due to fever/malaria [4].

### Ethics, consent and permissions

Written, informed consent was provided by the parent/guardian for all study participants. Ethics approval for the study was obtained from Comité d’Éthique du Nord Kivu (Université Catholique du Graben, ref 002/TEN/2012), the University of Alberta Human Research Ethics Board (ref Pro00055619), and regionally from the Médecin Chef de Zone, within the DRC Ministry of Health.

### Cross-sectional observational studies

In order to compare *Plasmodium falciparum* prevalence between displaced children and a control group, we selected the IDP camp of Bilobilo and the neighbouring village of Mubi. The camp consists of temporary housing structures built from tarpaulin and thatch covering an area of approximately 1 km^2^. It is located approximately 5 km from the village of Mubi along a road, which links North Kivu to Kisangani, a major city in Orientale province. The community of Mubi has a linear configuration, with households situated along the major road. The sites are at the same altitude, share the same climate, and both IDP camp residents and residents of Mubi village are ethnically Nande. Both the camp and Mubi are located within savannah-type ecology adjacent to a large and sparsely populated equatorial forest. Both the camp and village are also near the Lowa River, the main water source for its inhabitants. This area receives rainfall for 5 months during the wet season which is from November to March. We performed two cross-sectional observational studies: (1) community-based surveys of the IDP camp and the village; and (2) a survey of febrile children presenting to a health clinic that treats both IDP camp residents and village residents. At the time of the clinic survey, the median duration of displacement for the families living in the IDP camp was 8 months and at the time of the community-based survey the median duration of displacement for these families was 12.5 months.

Variables of interest were measured in a questionnaire that was administered to the parent or guardian of the participants, including: bed net ownership and use as potential protective factor, maternal education, household asset ownership and exposure to community violence. The RDT test used to diagnose *P. falciparum* can remain positive for up to a month after a malaria infection. To control for this, the questionnaire included items about recent febrile illness. In order to ensure exposure to mosquitoes was similar, participants in each study were sampled in the same time period.

### Community-based survey

The first survey was a community-based cross-sectional observational study. Community health workers, trained in malaria rapid diagnostic testing as previously described [17], visited a random sample of 200 temporary shelters within the Bilobilo IDP camp. Random sampling of temporary households in the camp was performed using a census created by non-governmental organizations providing services in the area. All households on the census were eligible for inclusion. For the control group, the community health workers partnered with a polio vaccination drive organized by the Ministry of Health to survey 200 households in the village of Mubi. Households were sampled using methodology modified from the Expanded Programme on Immunization (EPI) coverage survey method, World Health Organization (WHO) [18]. Because of a relatively linear arrangement of households along the principal road in the village, a random number, n, was generated and the nth household along the road was sampled. In each residence, one child under five was randomly selected and tested for *P. falciparum* infection using a HRP2-based rapid diagnostic test (Paracheck-Pf^®^ kit; Orchid Biomedical Systems, Goa, India). All participants who tested positive for *P. falciparum* were treated with the artemisinin-based combination therapy according to WHO recommendations [19].

A standard sample size calculation indicated that 199 subjects would be needed in each group in order to detect an absolute difference of 10 % in *P. falciparum* infection rates with a power of 80 % and the significance level of α = 0.05, assuming the community prevalence is 10 %.

### Clinic-based survey

The second survey was a clinic-based cross-sectional observational study. A convenience sample of 100 children presenting to the clinic for management of febrile illness from the IDP camp were tested for *P. falciparum* using the same HRP2-based rapid diagnostic test. Over the same time period, a convenience sample of 100 children (controls) from the village of Mubi presenting to the same clinic for management of febrile illness were enrolled. All participants who tested positive for *P. falciparum* were treated with artemisinin-based combination therapy according to WHO recommendations [19].

A standard sample size calculation indicated that 93 patients would be needed in each group in order to detect an absolute difference of 20 % in malaria infection rates, assuming a malaria prevalence of 50 % among febrile children presenting from the community; with a power of 80 % and a significance level of α = 0.05.

### Data analysis

For the community based survey, to examine the statistical association between the exposure, “displacement” (a binary variable—resident of IDP camp or community control), and the outcome, *P. falciparum* antigenemia (binary outcome—positive or negative), the Chi squared statistic or Fisher exact test was used, as appropriate. For the clinic based survey, to examine the statistical association between the exposure, displacement, and the outcome, malarial febrile illness, the Chi squared or Fisher exact test was used, as appropriate. For both surveys the Chi squared or Fisher exact test was used to examine the associations between the data collected in the questionnaire and displacement status.

In order to determine a wealth index, ownership of household assets were coded as binary variables and weighted using principal component analysis (PCA). This method, modified from Filmer and Pritchett [20] has been validated as a measure of household consumption and poverty and has been used in previous studies in sub-Saharan Africa (SSA) [21]. Eleven assets or household characteristics from the questionnaire were included to determine a wealth index; this index was then used to divide the cohort into wealth quintiles. The measures that we included were: household characteristics (electricity, brick vs mud and wattle construction) and asset ownership (bicycle, motor vehicle, radio, telephone, television, refrigerator, chicken, cow and goat).

Where multiple potential confounding variables were present, multivariable logistic regression models were used to determine independent predictors of important outcomes. Statistical analyses employed Stata 14.1 and SPSS Statistics version 19.

## Results

### Community-based survey

Two hundred children from the IDP camp and 200 from the control village were surveyed between July 15 and July 22, 2013 (see Additional file 2 for complete data set). Participant characteristics are shown in Table 1. Maternal education and household wealth were lower among families that live in the IDP camp (Table 1). Self-reported exposure to community violence among families living in the IDP camp was common including: theft (50 %), physical assault (9 %), sexual assault (5.5 %) and knife and gunshot injury (both at 0.5 %).

**Table 1:** Characteristics of participants in the community-based survey

	IDPs (Bilobilo) n = 200	Village controls (Mubi) n = 200	P value
Age^a^ (years): median (range)	2.6 (0.1-5)	2.4 (0.7–5)	0.11
Female sex^a^: n (%)	118 (59 %)	101 (51 %)	0.11
Number of children <5 in the household: median (range)	1 (1–4)	2 (1–4)	0.22
*Bed nets: n (%)*			
Household ownership	68 (34 %)	136 (68 %)	<0.001
Bed net use (index case)^a^	50 (25 %)	111 (56 %)	<0.001
*Maternal education: n (%)*			
No formal education	4 (2 %)	4 (2 %)	1.00
Primary	129 (65 %)	63 (32 %)	<0.001
Secondary	53 (27 %)	74 (37 %)	0.03
University	14 (7 %)	59 (30 %)	<0.001
*House construction: n (%)*			
Electricity	0	0	–
Brick	0	7 (3.5 %)	0.015
*Household assets: n (%)*			
Bicycle	45 (23 %)	98 (49 %)	<0.001
Motor vehicle	0	0	–
Radio	17 (8.5 %)	51 (25 %)	<0.001
Telephone	15 (7.5 %)	35 (18 %)	0.002
Television	0	0	–
Refrigerator	0	0	–
Chicken	41 (21 %)	112 (56 %)	<0.001
Cow	1 (0.5 %)	3 (1.5 %)	0.62
Goat	5 (2.5 %)	16 (8.0 %)	0.01
*Wealth quintile* ^*b*^ *: n (%)*			<0.0001
Poorest	108 (54 %)	42 (21 %)	
Second	34 (17 %)	24 (12 %)	
Middle	26 (13 %)	27 (14 %)	
Fourth	19 (9.5 %)	44 (22 %)	
Richest	13 (6.5 %)	63 (32 %)	
*P. falciparum infection (RDT positive)* ^*a*^: n (%)	35 (17.5 %)	15 (7.5 %)	0.009

^a^Age, sex, bed net use, and *P. falciparum* infection refer to the index child; all other measures refer to the family/household

^b^Based on principal component analysis of household wealth indicators [33]. For four of the eleven indicators, no participant in the village or camp owned the asset (electricity in the house, vehicle, television and refrigerator); these indicators and they did not contribute to the wealth index

**Table 2:** Characteristics of participants in the clinic-based survey of children with fever presenting for care

	IDPs (Bilobilo) n = 100	Village controls (Mubi) n = 100	P value
Age^a^ (years): median (range)	2.6 (1–5)	3 (1–5)	0.49
Female sex^a^: n (%)	47 (47 %)	53 (53 %)	0.48
*Bed nets: n (%)*			
Household ownership	21 (21 %)	75 (75 %)	<0.001
Bed net use^a^ (index case)	16 (16 %)	65 (66 %)	<0.001
*Maternal education: n (%)*			
No formal education	1 (1 %)	0	1.00
Primary	29 (29 %)	13 (13 %)	0.009
Secondary	50 (50 %)	42 (42 %)	0.32
University	20 (20 %)	45 (45 %)	<0.001
*House construction: n (%)*			
Electricity	0	0	–
Brick	0	4 (4 %)	0.12
*Household assets: n (%)*			
Bicycle	45 (45 %)	75 (75 %)	<0.001
Motor vehicle	1 (1 %)	1 (1 %)	1.00
Radio	21 (21 %)	55 (55 %)	<0.001
Telephone	11 (11 %)	41 (41 %)	<0.001
Television	1 (1 %)	2 (2 %)	1.00
Refrigerator	0	0	–
Chicken	60 (60 %)	59 (60 %)	1.00
Cow	12 (12 %)	20 (20 %)	0.13
Goat	38 (38 %)	39 (39 %)	0.88
*Wealth quintile* ^*b*^ *: n (%)*			<0.001
Poorest	29 (29 %)	11 (11 %)	
Second	28 (28 %)	12 (12 %)	
Middle	20 (20 %)	20 (20 %)	
Fourth	16 (16 %)	24 (24 %)	
Richest	7 (7 %)	33 (33 %)	
*Exposure to community violence: n (%)*			
Theft	59 (59 %)	12 (12 %)	<0.001
Physical assault	15 (15 %)	1 (1 %)	<0.001
Sexual assault	22 (22 %)	1 (1 %)	<0.001
Knife injury	1 (1 %)	0	1.00
Gunshot	2 (2 %)	0	0.50
*P. falciparum* infection^a^ (RDT positive): n (%)	78 (78 %)	39 (39 %)	<0.001

^a^Age, sex, bed net use, and *P. falciparum* infection refer to the index child; all other measures refer to the family/household

^b^Based on principal component analysis of household wealth indicators. For two of the eleven indicators (electricity in the house and refrigerator), no participant in the village or camp owned the asset; these indicators did not contribute to the wealth index

**Fig. 1:**
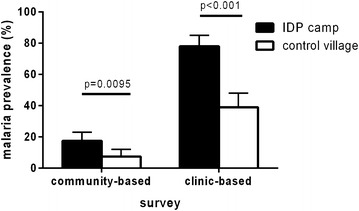
Higher burden of *P. falciparum* among children living in an IDP camp (*black bars*), compared to a neighbouring village (*white bars*). Results are based on community-based surveys of the IDP camp at Bilobilo (n = 200) and the neighbouring village of Mubi (n = 200), as well as a clinic-based survey of febrile children from the IDP camp (n = 100) and the village (n = 100). The prevalence of *P. falciparum* infection in the community and among febrile children attending a health clinic was significantly higher among IDP camp residents than village controls

The point-prevalence of *P. falciparum* infection was 35/200 (17.5 %) among IDPs and 15/200 (7.5 %) for the control group (OR 2.6; 95 % CI 1.3–4.1; P = 0.0095) (Fig. 1). Of note, these children were minimally symptomatic and had not presented for medical care, but were actively identified in the community at large. Because RDT positivity may persist for up to 1 month after successful treatment of infection [22], this comparison was repeated after exclusion of participants with a history of febrile illness within the past month, and found similar results (OR 3.0; 95 % CI 1.4–6.3; P = 0.003). Factors associated with *P. falciparum* infection in the cohort included bed net ownership: *P. falciparum* was detected in 18/202 (8.9 %) children from households owning a bed net compared to 32/195 (16 %) without (OR 0.50; 95 % CI 0.27–0.92; P = 0.024). Age, sex, household size (number of children under five), maternal education, and household wealth were not statistically significantly associated with *P. falciparum* infection in the cohort. In a multi-variable logistic regression model accounting for possible confounding between covariates, IDP camp residence remained the only significant independent predictor of *P. falciparum* positivity (aOR 2.6; 95 % CI 1.2–5.7; P = 0.013).

**Fig. 2:**
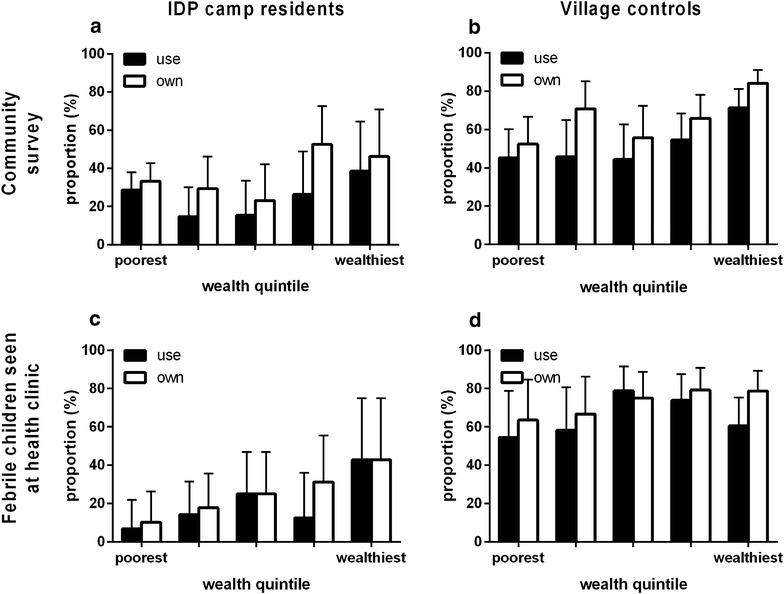
Bed net ownership (*white bars*) and use (*black bars*) in a Congolese IDP camp and neighbouring village. Self-reported household bed net ownership and use by an index child <5 in the household the night prior to the survey, according to wealth quintile. Results are based on community-based surveys of the IDP camp at Bilobilo (**a**, n = 200) and the neighbouring village of Mubi (**b**, n = 200), as well as a clinic-based survey of febrile children from the IDP camp (**c**, n = 100) and the village (**d**, n = 100). In multivariable logistic regression models, IDP camp residence (relative to control village) and wealth index were independent predictors of bed net ownership

Household bed net ownership was lower in the IDP camp compared to the controls (34 vs 68 %, P < 0.001). Bed net use by the index child the night prior to the survey was also lower among the IDPs than controls (25 vs 56 %, P < 0.001, Fig. 2). Factors associated with bed net ownership in the cohort included higher household wealth index; 59/76 (78 %) of the households from the wealthiest quintile owned a bed net compared to 58/150 (39 %) of households in the poorest quintile (P < 0.001). Maternal education was not associated with bed net ownership (P = 0.242) or use (P = 0.207).

### Clinic-based survey

Between January 5 and February 16, 2013, 100 children from the IDP camp and 100 children from the control village presenting for management of febrile illness were included (see Additional file 3 for complete data set). Clinical characteristics, listed in Table 3, show that severity of malaria among children from the IDP camp was similar or greater than control children from the village. As in the community-based survey, significant differences in household wealth and maternal education were observed among IDP camp residents. Comparison of exposure to community violence also showed significant differences between the two groups, with theft, physical and sexual assault higher among displaced families (Table 2).

**Table 3:** Clinical characteristics of children presenting to the clinic for treatment of febrile illness

Clinical characteristics	Malarial febrile illness			Non-malarial febrile illness		
	IDP	Village	P value	IDP	Village	P value
Seizure	18 (23 %)	8 (21 %)	0.75	4 (18 %)	13 (21 %)	0.76
Coma	1 (1.3 %)	0 (0 %)	0.48	1 (4.5 %)	3 (4.9 %)	0.94
Anaemia	10 (13 %)	4 (10 %)	0.70	4 (18 %)	5 (8.2 %)	0.20
Trouble breathing	51 (65 %)	15 (39 %)	0.006	13 (59 %)	44 (72 %)	0.26

The prevalence of malaria among febrile children from the IDP camp was 78/100 (78 %) compared to 39/100 (39 %) for the village controls (OR 5.5; 95 % CI 3.0–10.3; P < 0.001). To adjust for potential false-positive test results due to recent infection, this comparison was repeated after exclusion of children with a history of recent febrile illness, with similar results (OR 6.5; 95 % CI 3.1–13.6; P < 0.001). Factors associated with malaria infection in the clinic-based cohort included bed net ownership and use. Malaria was detected less frequently in children from households owning a bed net (39/96 (41 %) vs 77/103 (75 %); OR 0.23; 95 % CI 0.13–0.42; P < 0.001), and less frequently if the child was reported to sleep under the bed net (28/81 (35 %) vs 87/116 (75 %); OR 0.18; 95 % CI 0.095–0.33; P < 0.001). Age, sex, household size (number of children under 5), maternal education, and household wealth were not statistically significantly associated with malaria infection in the cohort. In a multivariable model adjusting for potential confounding effects, IDP camp residence was an independent risk factor (aOR 2.7; 95 % CI 1.1–6.6; P = 0.027) and sleeping under a bed net was an independent protective factor (aOR 0.25; 95 % CI 0.10–0.60; P = 0.002) for malaria infection.

Bed net ownership and use were significantly lower for the children from the IDP camp compared to the children from the village (21 vs 75 %, P < 0.001, and 16 vs 65 %, P < 0.001), respectively (Fig. 2). As in the community-based survey, higher household wealth and maternal education were associated with higher rates of bed net ownership (Fig. 2, P < 0.01 for all comparisons).

## Discussion

The primary objective of this study was to compare the burden of malaria among children under five living in an IDP camp to a control group of children under five living in a nearby village, with similar environmental (geography, altitude, climate) and genetic characteristics. In both a community-based survey of minimally symptomatic children and a survey of febrile children presenting for care at a health clinic, the point prevalence of *P. falciparum* was significantly higher among children living in an IDP camp than neighbouring village controls. Previous studies have found that the physical and psychological health of displaced populations is substantially poorer than non-displaced populations [6]. The present study is noteworthy in describing a clear association between population displacement and malaria, a leading cause of childhood mortality [23].

A notable strength of this report is the convergent results from two independent surveys addressing two manifestations of *P. falciparum* infection: minimally symptomatic infection in the community and febrile illness presenting to a clinic for care. In both surveys, IDP camp residence was reproducibly associated with *P. falciparum* infection, with and without adjustment for recent febrile illness, and remained a significant independent predictor in multivariable logistic regression models. The consistency of this association lends strength to these conclusions. Possible causal pathways linking displacement to *P. falciparum* infection were explored, and bed net ownership and use appeared to be protective against malaria infection in this cohort, as in past reports [24]. Furthermore, bed net ownership was significantly lower in the IDP camp compared to village control households. Household wealth index was a determinant of bed net ownership in both the IDP camp and the neighbouring village, consistent with previous reports [25–27]. These findings paint a plausible and consistent picture: poverty exacerbated through displacement, reduced access to and/or use of bed nets as a prevention measure, and higher rates of malaria infection among children living in IDP camps.

A recent meta-analysis of the prevalence of *P. falciparum* infection in children under five, collected via community surveys, shows a wide range in prevalence from 0.4 to 78 % (median 19 %) in SSA [28]. A study examining the prevalence of *P. falciparum* among residents of an IDP camp in Uganda found a prevalence of parasitaemia of 11 % [13]. The prevalence in the present study (18 % in the IDP camp and 7.5 % in the control village) is similar, suggesting that the setting of this study was representative of other tropical African environments. Studies of children with febrile illness in SSA show a prevalence of malaria in the range from 26 to 49 % [29–32]. Another study examining febrile patients from a refugee camp in Kenya described a malaria prevalence of 50 % [33]. In the present study, the malaria prevalence among febrile children from the village (39 %) falls within this range; however the prevalence of malaria in febrile children from the IDP camp (78 %) is substantially higher, illustrating the elevated burden of malaria in this group.

Numerous environmental and biological factors may increase the risk of *P. falciparum* infection among IDP camp residents. Common risks for vector exposure include standing water or other mosquito breeding grounds and exposure to night-biting mosquitoes caused by lack of shelter and/or lack of a bed net to sleep under. This study found a large discrepancy in bed net ownership and use between children living in the IDP camp and children living in the village. These results are surprising because humanitarian agencies working in the region distribute bed nets to families living in the IDP camp. Future directions of this research will include an exploration of reasons for under-utilization of bed nets among IDP camp residents in future studies. Children living in IDP camps may be at risk for other common childhood diseases, such as pneumonia, diarrhoea, and malnutrition [34], due to suboptimal living conditions [35, 36]. Indeed, in the present study, non-malarial febrile illness associated with respiratory distress (i.e., clinical pneumonia) was a common cause of presentation to the clinic (59 % of IDP and 72 % of control patients).

The description of household asset ownership, maternal education, and exposure to community violence among IDP camp households provides new data in this neglected group and demonstrates consistently, in both community and clinic-based surveys, a level of severe deprivation, even relative to an already resource-limited rural African community. While household asset depletion due to displacement may be an intuitive finding, lower maternal education in the IDP camp may relate to easier access to safe transportation and accommodation among higher educational strata. Socio-economic risk factors for malaria include poverty and lower maternal education level [37]. The mothers in the IDP camps were also less likely to have higher education compared to mothers living in the village, this is important as maternal education has a known impact on child health [37].

This study has several limitations. First, the cross-sectional design and limited sampling time frame provided estimates of point-prevalence, but disease incidence was not measured. The ability to draw conclusions about causality is limited because the exposure and outcome measures are taken at the same time so there is no information about the temporal relationship. Second, rapid diagnostic tests were used to identify *P. falciparum* infection in the cohort. The HRP-2 RDT may have limited sensitivity at low parasite density, which may have led to underestimation of *P. falciparum* prevalence, particularly in the community-based surveys of minimally symptomatic children [38, 39]. The use of additional diagnostic modalities (microscopy and PCR) would be desirable to more fully describe the parasite burden. A single IDP camp and control village were compared, whereas a study with multiple camps and control sites would provide more robust and generalizable data. Bed net use was measured by parental report for the previous night, whereas children need to be consistently sleeping under the bed net to have constant coverage and protection from malaria.

## Conclusions

This study showed that displaced children under five appear to be at higher risk for malaria infection than neighbouring village controls in the DRC. Current control measures that focus on bed net distribution may be under-utilized and appear insufficient to limit childhood malaria. Additional targeted control measures, such as indoor residual spraying and/or active case finding and treatment of minimally symptomatic individuals, should be considered. Malaria remains a major cause of childhood mortality in the tropics, and children living in IDP camps may represent a vulnerable population bearing a disproportionate burden of illness.

## Additional files

10.1186/s12936-016-1479-z Map showing A. Democratic Republic of Congo (DRC) within Africa, B. North Kivu within the DRC, and C. Location of study sites Mubiand Bilobilo within North Kivu and relative to the provincial capital, Goma.
10.1186/s12936-016-1479-z. SPSS file providing data for clinic-based survey.
10.1186/s12936-016-1479-z. SPSS file providing data for community-based survey.

## Abbreviations

ACT: artemisinin combination therapy
aOR: adjusted odds ratio
EPI: Expanded Programme On Immunization
DRC: Democratic Republic of the Congo
HRP: histidine-rich protein
IDP: internally displaced persons
OR: odds ratio
PCA: principal component analysis
RDT: rapid diagnostic test
SSA: sub-Saharan Africa
WHO: World Health Organization
