# Supplementary material for: Burden of malaria is higher among children in an internal displacement camp compared to a neighbouring village in the Democratic Republic of the Congo
Source: Malar J. 2016 Aug 25;15(1):431. doi: 10.1186/s12936-016-1479-z (PMC4997722; doi:10.1186/s12936-016-1479-z)
Supplement: Supplementary file 1 — 10.1186/s12936-016-1479-z Map showing A. Democratic Republic of Congo (DRC) within Africa, B. North Kivu within the DRC, and C. Location of study sites Mubiand Bilobilo within North Kivu and relative to the provincial capital, Goma. [file 12936_2016_1479_MOESM1_ESM.pdf]

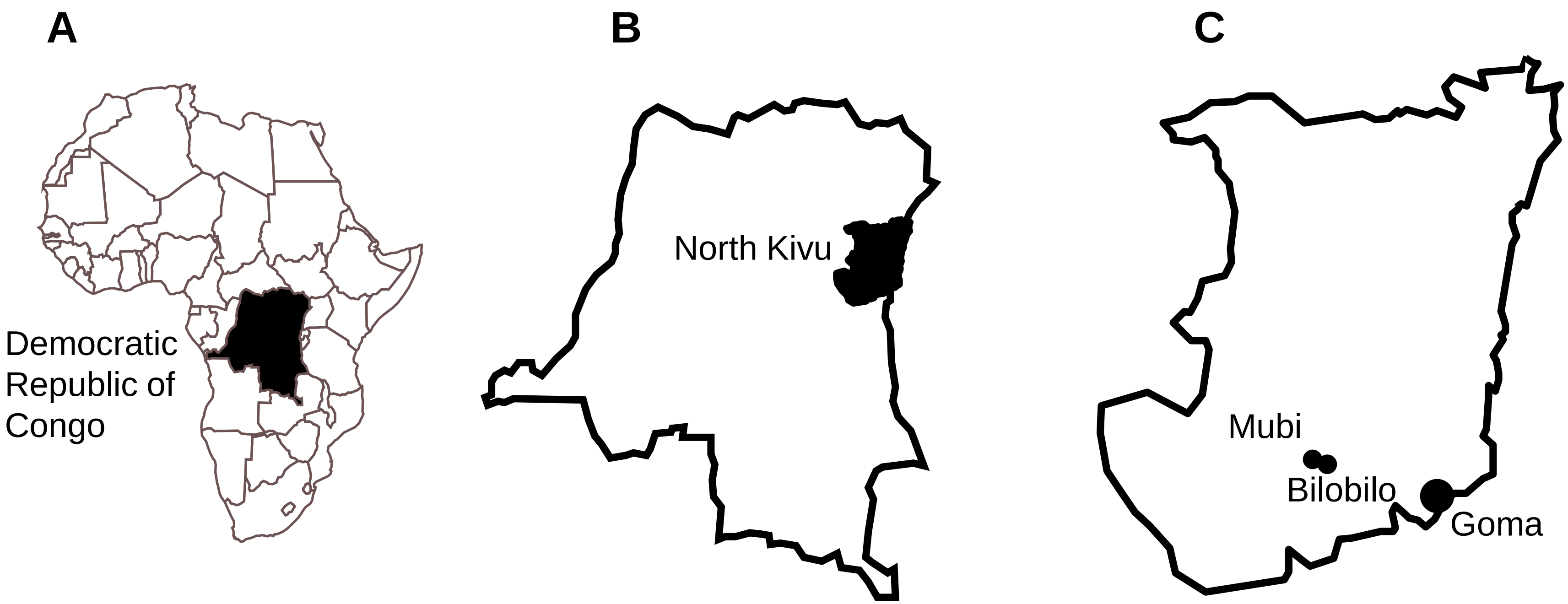

Supplemental Figure 1. Map showing A. Democratic Republic of Congo (DRC) within Africa, B. North Kivu within the DRC, and C. Location of study sites Mubi and Bilobilo within North Kivu and relative to the provincial capital, Goma.
